# Evidence of Viremia in Dairy Cows Naturally Infected with Influenza A Virus, California, USA

**DOI:** 10.3201/eid3107.250134

**Published:** 2025-07

**Authors:** Jason Lombard, Chloe Stenkamp-Strahm, Brian McCluskey, Blaine Melody

**Affiliations:** Colorado State University, Fort Collins, Colorado, USA (J. Lombard, C. Stenkamp-Strahm, B. McCluskey); Lander Veterinary Clinic, Turlock, California, USA (B. Melody)

**Keywords:** influenza, viruses, respiratory infections, zoonoses, viremia, cattle, influenza A virus, California, USA

## Abstract

We confirmed influenza A virus (IAV) by PCR in serum from 20 cows on 3 affected dairy farms in California, USA. Our findings indicate the presence of viremia and might help explain IAV transmission dynamics and shedding patterns in cows. An understanding of those dynamics could enable development of IAV mitigation strategies.

In March 2024, the US Department of Agriculture’s National Veterinary Services Laboratories (NVSL) confirmed avian influenza virus (IAV) A(H5N1) clade 2.3.4.4b in dairy cows in Texas, USA (*1*,*2*). That subtype was further characterized as genotype B3.13. Since that detection, >1,070 herds in 17 states have been affected; most of those herds are in California (*3*). Clinical signs observed have been variable, but fever, nasal discharge, loss of milk production, and mastitis are common (*4*).

Experimental and field investigations into the transmission dynamics, pathogenesis, and epidemiology of H5N1 virus in cows are ongoing. Researchers have inoculated cows via the intramammary route in 3 studies, and resulting clinical signs were similar to those from field reports of affected cows, including severe disease requiring euthanasia. Viral RNA was found in milk samples in all studies (*5*–*7*) but in blood samples in only 1 study (*7*). Early in the ongoing outbreak, nasal swab, whole blood, serum, and milk samples were collected from affected dairies in Texas, New Mexico, Kansas, and Ohio. Viral RNA was detected in nasal swab (10/47 cows), whole blood (3/25 cows), serum (1/15 cows), and milk (129/192 cows) samples (*4*).

We sampled cows using a serial sampling design early in the outbreak on affected dairy farms in California. We report detection of IAV RNA in serum samples from lactating dairy cows.

## The Study

In collaboration with a private veterinary practice, daily bulk tank milk (BTM) surveillance for IAV was established in 19 initially unaffected dairies in California; the dairies were in a geographic area that had not experienced IAV infection. BTM samples were collected and transported to the veterinary practice’s in-house laboratory for testing for IAV by real-time reverse transcription PCR (RT-PCR). When positive results were detected, a National Animal Health Laboratory Network (NAHLN) laboratory performed confirmation testing, and NVSL confirmed the viral subtype, clade and genotype (H5N1, 2.3.4.4b, B3.13) of virus circulating in each herd. One dairy farm was dropped from surveillance because of inconsistent sampling.

During October 24–November 28, 2024, all 18 herds had IAV detected. For 5 of those herds, we initiated individual cow sampling soon after BTM detection. Individual cow samples were tested at the NAHLN laboratory using the same methods. We considered cycle threshold (Ct) values of <40 to have detectable viral RNA. For Ct values of 38–40 that were initially considered suspect, we retested and considered them positive if Ct values remained <40. The objective was to collect a battery of samples and cow-level information to investigate viral transmission dynamics, viral shedding, and other factors that might lead to development of disease mitigation strategies on affected dairies.

For the 5 dairies where we conducted individual cow sampling, the sampling plan called for collection of nasal swab, serum, urine, and milk (when applicable) samples from preweaning heifers, nonlactating cows, cows that had recently calved, and sick and healthy lactating cows. Determination of sick versus healthy cows varied by farm; we used results of pen milk sampling, dairy management using clinical signs, and biometric data, when available. For the purposes of this study, we focused on testing serum from lactating cows to establish the presence of viremia. Overall, 3 of 5 farms had cows with detectable viral RNA in serum; samples were taken from 144 cows (Table). Although individual cows were sampled at multiple time points, IAV was detected in serum at a single sampling period.

**Table:** Summary of influenza A virus testing where viremia was detected by PCR in cows from 3 affected dairy herds in California, USA*

Herd	Breed	No. lactating cows sampled	No. sampling periods	No. (%) lactating cows with RT-PCR–positive serum samples	Interval for each sampling period, d		No. serum samples collected
					Onset of clinical signs to positive serum samples	First bulk tank milk detection to positive serum samples	
A	Jersey	40	2	11 (27.5)	3, 10	14, 21	80
B	Holstein	74	3	5 (6.8)	3, 9, 13	5, 11, 15	107
C	Holstein	30	1	4 (13.3)	5	14	30
Total		144		20 (13.9)			217

*RT-PCR, reverse transcription PCR.

For the 3 herds with serum RT-PCR IAV detection, we collected samples during 1 to 3 sampling periods within 21 days of BTM detection and within the first 13 days from onset of clinical signs (Figure). Of the 20 cows with serum detection, all cows had virus detected in milk at the same sampling or within 24 hours, whereas 4 (20%) also had concurrent detection in urine. We did not detect virus concurrently in any nasal swab samples. Ct values from serum ranged from 32.3 to 39.2, from milk ranged from 14.4 to 28., and from urine ranged from 28.5 to 38.7. The prevalence of viremia as a percentage of cows sampled per herd ranged from 6.8% to 27.5%; the Jersey herd (herd A) had the highest prevalence.

**Figure:**
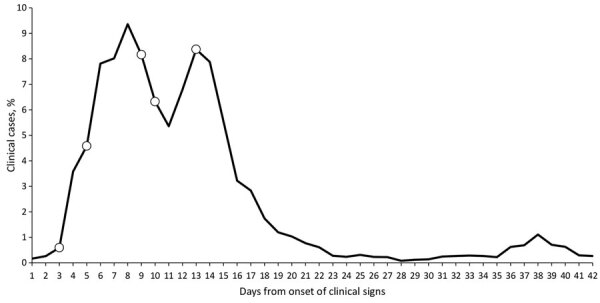
Epidemic curve for clinical cases of influenza A virus in 3 affected dairy herds in California, USA. We averaged the percentage of daily cases over the 3 farms and then calculated a rolling 3-day average. Open white circles on the curve represent days that virus was detected in serum.

## Conclusions

Our results suggest that a percentage of lactating cows on dairies affected by H5N1 virus experience viremia before or during the peak of clinical cases in the herd. We detected viral RNA in serum of each PCR-positive cow at a single sample date. Viremia therefore appears to be transient, but the duration is unknown because cows were not sampled daily. Although the finding of viremia does not specify how IAV made it to the bloodstream, virus present in circulation suggests that multiple exposure pathways might be possible, including oral and respiratory routes. Intramammary inoculation studies have shown viral RNA to be in multiple tissues at necropsy (*6*,*7*), although viremia had not been consistently detected.

Viremia enables virus to reach many tissues in the body, including the kidneys, which is evident in this study given detectable RNA in urine samples. That process raises concern for food safety and whether viremia could lead to the presence of H5N1 virus in meat from culled dairy cows. A study that evaluated condemned carcasses found viral RNA in 1 of 109 total samples (*8*). Further, an aborted fetus from farm B, not from a known viremic cow, was positive for H5N1 virus in lung and brain tissue by PCR and immunohistochemical staining. H5N1 virus can move into the reproductive tract and is associated with abortion, which also has implications for the use of fetal serum products. All cows in this study had IAV detection in serum and milk, so it is unclear whether intramammary infection led to viremia or viremia led to intramammary infection. Three cows classified as healthy had viral RNA detected in serum, and 1 of the 3 had viral RNA detected in serum and urine. The relationship between viremia and clinical signs is therefore unclear, although we might have sampled those cows before the onset of clinical disease.

Determining whether the viremia we detected is a rare event is crucial. Viremia has only been detected in 1 previous H5N1 intramammary experimental infection study and 1 field study. To clarify the frequency of viremia, more studies that evaluate IAV RNA in the serum of cattle should be completed. The prevalence of viremia detected in the Jersey breed herd compared with the 2 Holstein breed herds suggests breed differences in susceptibility to viremia from IAV infection might be involved, but differences in cow selection by farm might have affected prevalence. We also recommend a genetic comparison of viral strains collected between studies and between states. Viremia in California dairy cows could be the result of viral evolution, because viremia was not well documented previously, and experimental studies used a strain of H5N1 virus from the early stages of this outbreak. Further in-depth studies that include viral sequencing are necessary to strengthen the evidence supporting our conclusion.

In summary, findings of IAV in serum of cows on farms in California indicates the presence of viremia and could help explain viral transmission dynamics and shedding patterns in cows. Understanding such dynamics could help in development of mitigation strategies to prevent transmission and spread of IAVs, including H5N1 virus.
